# Automatic detection of ventilatory modes during invasive mechanical ventilation

**DOI:** 10.1186/s13054-016-1436-9

**Published:** 2016-08-14

**Authors:** Gastón Murias, Jaume Montanyà, Encarna Chacón, Anna Estruga, Carles Subirà, Rafael Fernández, Bernat Sales, Candelaria de Haro, Josefina López-Aguilar, Umberto Lucangelo, Jesús Villar, Robert M. Kacmarek, Lluís Blanch

**Affiliations:** 1 Clínica Bazterrica y Clínica Santa Isabel, Departamento de Ciencias Fisiológicas, Farmacológicas y Bioquímicas, Facultad de Medicina, Universidad Favaloro, Buenos Aires, Argentina; 2 CIBER de Enfermedades Respiratorias, Instituto de Salud Carlos III, Madrid, Spain; 3 Parc Taulí Hospital Universitari, Institut d’Investigació i Innovació Parc Taulí (I3PT), Universitat Autònoma de Barcelona, c. ParcTaulí 1, 08208 Sabadell, Spain; 4 Servei de Medicina Intensiva, Hospital Sant Joan de Deu-Fundació Althaia, Universitat Internacional de Catalunya, Manresa, Spain; 5 Better Care, Barcelona, Spain; 6 Department of Perioperative Medicine, Intensive Care and Emergency, Cattinara Hospital, Trieste University, Trieste, Italy; 7 Research Unit, Hospital Universitario Dr. Negrín, Las Palmas de Gran Canaria, Spain; 8 Department of Respiratory Care, Massachusetts General Hospital and Department of Anesthesiology, Harvard Medical School, Boston, MA USA

**Keywords:** Mechanical ventilation, Automatic detection, Ventilatory mode, Information systems in critical care

## Abstract

**Background:**

Expert systems can help alleviate problems related to the shortage of human resources in critical care, offering expert advice in complex situations. Expert systems use contextual information to provide advice to staff. In mechanical ventilation, it is crucial for an expert system to be able to determine the ventilatory mode in use. Different manufacturers have assigned different names to similar or even identical ventilatory modes so an expert system should be able to detect the ventilatory mode. The aim of this study is to evaluate the accuracy of an algorithm to detect the ventilatory mode in use.

**Methods:**

We compared the results of a two-step algorithm designed to identify seven ventilatory modes. The algorithm was built into a software platform (BetterCare® system, Better Care SL; Barcelona, Spain) that acquires ventilatory signals through the data port of mechanical ventilators. The sample analyzed compared data from consecutive adult patients who underwent >24 h of mechanical ventilation in intensive care units (ICUs) at two hospitals. We used Cohen’s kappa statistics to analyze the agreement between the results obtained with the algorithm and those recorded by ICU staff.

**Results:**

We analyzed 486 records from 73 patients. The algorithm correctly labeled the ventilatory mode in 433 (89 %). We found an unweighted Cohen’s kappa index of 84.5 % [CI (95 %) = (80.5 %: 88.4 %)].

**Conclusions:**

The computerized algorithm can reliably identify ventilatory mode.

**Electronic supplementary material:**

The online version of this article (doi:10.1186/s13054-016-1436-9) contains supplementary material, which is available to authorized users.

## Background

Monitoring is one of the main reasons for admission to intensive care units (ICUs). Up to 77 % of admissions to medical ICUs take place, at least in part, for monitoring purposes, even though only 10 % of the patients only monitored will subsequently have indications for major interventions [1]. Accordingly, huge investments in monitoring technology have led to the development of a wide array of monitoring devices (bedside monitors, mechanical ventilators, special devices, etc.) that generate large quantities of data. However, these data are underexploited for two main reasons. First, many data are typically presented only fleetingly on screens that clinicians see only when they are at the patient’s bedside. So, unless an alarm is triggered, hours of acquired data are lost [2]. Trended data is of little use in identifying asynchronies since asynchronies can occur sporadically and need to be identified as they occur, and displayed as occurring at a frequency over time. Trending data is most useful in identifying physiologic change not short-term events. Second, data that is not interpreted are useless; to become useful, data must become information, by being processed, organized, structured, and contextualized [3]. Valuable information has often remained buried even when the data in which it is based were widely available. For instance, invasive monitoring of arterial blood pressure has been used since the beginnings of ICUs, but in recent years the analysis and processing of these data has yielded information far more valuable than arterial blood pressure alone. For example, pulse contour can be used to estimate cardiac output [4, 5], and pulse pressure variation can predict the cardiovascular system’s response to a fluid load [6–8]. Tools to transform data from mechanical ventilators into meaningful information should help critical care clinicians anticipate harmful events, and expert systems could help them solve problems quickly and obtain expert advice.

The Better Care® software platform (Better Care S.L., Barcelona, Spain) processes (standardizes, resamples, synchronizes, analyzes, and stores) data from the data ports of mechanical ventilators or other monitoring devices [9]. It has a set of algorithms that evaluate the behavior of airway pressure and flow to automatically detect potentially harmful events (ineffective inspiratory efforts, double triggering, airway secretions, short and long cycles, aborted inspirations). In a previous study assessing the prevalence and time course of asynchronies throughout mechanical ventilation, we found a median of 3.41 % asynchronies in all patients [10]. However, that study probably underestimated the magnitude of the problem because many of the analyses were limited to the expiratory part of the flow tracing, where the shape of the curve does not depend on the ventilatory mode. Most device communication protocols do not provide information about the ventilatory mode in use, and different manufacturers use different, often meaningless, names for identical modes [11, 12]. For a system to detect events during inspiration, it must be able to identify the ventilatory mode. A system that could identify the ventilatory mode would also enable protocols to facilitate communication between medical devices and expert systems by converting data from proprietary device languages to standardized formats guaranteeing interoperability [13]. The aim of the present study is to validate an algorithm to identify the most prevalent ventilatory modes based on the analysis of airflow and airway pressure waveforms.

## Methods

### Software

The Better Care® system interacts directly with the signal output of medical devices through a device-specific connection driver. Mechanical ventilators and bedside monitors are connected to the system using an ED41000P2-01 remote access server (Lantronix, Irvine, CA, USA). The system standardizes the signals, associating each recorded curve with the parameter it represents, translates proprietary names into standard names, and resamples signals to a frequency of 200Hz. Standardized signals are then analyzed, tagged, converted to Digital Imaging and Communication in Medicine (DICOM) format, and stored in the hospital’s picture archiving and communication system (PACS).

### Setting

The study took place in two general ICUs (Parc Taulí University Hospital, Sabadell, Spain and Hospital Sant Joan de Deu-Fundació Althaia, Manresa, Spain) with 18 beds (4 beds in one hospital and 14 in the other) equipped with the Better Care® platform and one of the following ventilators: Evita 4 (Dräger, Lübeck, Germany), Puritan Bennet 840 (Covidien, Plymouth, MN, USA), or Servo I (Maquet, Fairfield, NJ, Sweden). The institutional review board of the Parc Tauli University Hospital approved the protocol and waived informed consent because the study was non-interventional, posed no added risk to the patient, and did not interfere with usual care.

### Patients

We studied consecutive patients aged >18 years admitted to one of the equipped beds who underwent mechanical ventilation for >24 hours.

### Protocol

A team composed of two nurses and one physician who were not involved in any clinical decisions recorded the ventilatory mode at the bedside once a day; the resulting log (including a timestamp for each entry) was used as a gold standard against which the Better Care® system’s automatic assignments were compared.

The Better Care® software recorded and analyzed airway pressure and flow waveforms and calculated tidal volumes for every breath during the hour preceding the team’s recording of ventilatory mode and applied a two-step algorithm to determine the specific ventilatory mode. The first step analyzes each breath and classifies it in one of seven categories according to the behavior of inspiratory time (TI)s, flow, airway pressure (P_AW_), and tidal volume (VT) (Table 1). To assess the stability of the different parameters, the system calculates a variability index (VI) as follows:

$$ \mathrm{V}\mathrm{I}=\sqrt{\frac{{\left(\mathrm{Actual}\kern0.24em \mathrm{Value}-\mathrm{Mean}\kern0.24em \mathrm{Value}\right)}^2}{\mathrm{Mean}\kern0.24em \mathrm{Value}}}\times 100 $$

where “Actual Value” is the measured value for a given variable and “Mean Value” is the running mean value for the same variable over the last 20 breaths (even though step 1 of the algorithm is breath-based, the stability of a given parameter is evaluated in the context of the preceding breaths). In other words, VI represents the variation in the parameter as a percentage of the mean value for the last 20 breaths. A variable was considered to be constant if the VI was less than 10 %.

**Table 1:** Breath classification strategy

	Breath characteristics						
	TI	Flow	Flow Slp	P_AW_	P_AW_ lev	Volume	P_300_
Type1	V	V	V	V	1	V	No
Type2	C	C	C	V	2	C	No
Type3	C	V	C	V	2	C	No
Type4	C	V	V	C	2	V	No
Type5	V	V	V	C	2	V	No
Type6	V	V	V	V	2	V	Yes
Type7	V	V	V	V	2	V	No

*Abbreviations*: *TI* inspiratory time, *Flow* inspiratory flow, *Flow Slp* inspiratory flow slope, *P*_*AW*_ peek airway pressure, *P*_*AW*_ *lev* number of PAW levels, *Volume* tidal volume, *P*_*300*_ 300 msec pause between inspiratory an expiratory time, *C* constant, *V* variable

The second step identifies the ventilator mode in proportion of the breaths classified into each category in the hour being analyzed (Table 2): continuous positive airway pressure (CPAP) (at least 90 % of breaths are classified as Type 1); volume control-continuous mandatory ventilation or volume-controlled ventilation with constant flow (VC-CMV) (at least 90 % of breaths are classified as Type 2: volume control-continuous mandatory ventilation with decelerating flow or volume control ventilation with decelerated flow (VC-CMV_DF_) (at least 90 % of breaths are classified as Type 3); pressure control-continuous mandatory ventilation or pressure-controlled ventilation (PC-CMV) (at least 90 % of breaths are classified as Type 4); pressure control-continuous spontaneous ventilation or pressure support ventilation (PC-CSV) (at least 90 % of breaths are classified as Type 5); spontaneous proportional assist or proportional assist ventilation (PC-CSV_R_) (which also includes neurally adjusted ventilatory assist, NAVA) (at least 80 % of breaths are classified as Type 6 and no Type 7 breaths are present); spontaneous proportional assist or proportional assist ventilation + (PC-CSV_R_+) (at least 80 % of breaths are classified as Type 6 or Type 7).

**Table 2:** Mode classification strategy

	Breath type						
	1	2	3	4	5	6	7
CPAP	>80 %						
VC-CMV		>80 %					
VC-CMV_DF_			>80 %				
PC-CMV				>80 %			
PC-CSV					>80 %		
PC-CSV_R_+						>90 %	
PC-CSV_R_						0 %	>90 %

*Abbreviations*: *CPAP* continuous positive airway pressure, *VC-CMV* volume control-continuous mandatory ventilation or volume-controlled ventilation with constant flow, *VC-CMV*_*DF*_ volume control-continuous mandatory ventilation with decelerated flow; or volume-controlled ventilation with decelerated flow, *PC-CMV* pressure control-continuous mandatory ventilation or pressure-controlled ventilation, *PC-CSV* pressure control-continuous spontaneous ventilation or pressure support ventilation, *PC-CSV*_*R*_ spontaneous proportional assist or proportional assist ventilation, *PC-CSV*_*R*_ *+* spontaneous proportional assist or proportional assist ventilation +

If the proportion of breaths classified did not fall into one of the above categories, the system labeled the record as other modes. This label encompassed modes beyond the scope of this algorithm, records that the system was unable to correctly classify, and hours in which the ventilatory mode changed.

### Statistical analysis

We used the unweighted Cohen’s kappa coefficient to assess the agreement between the team’s recordings in the log (gold standard) and the Better Care® mode detection algorithm.

## Results

The team recorded the ventilatory mode 486 times (Sabadell: n = 301; Manresa: n = 185) in 73 patients (Sabadell: n = 31; Manresa: n = 42). The algorithm correctly labeled 433 (89 %) hours of ventilation mode (Table 2). The unweighted Cohen’s kappa coefficient was 84.5 % [95 % CI: 80.5–88.4 %)].

The system labeled 56 (12 %) records as other modes; this label was correct in 23 cases (5 % of the total) because the mode did not fit any of the studied modes. The system mislabeled a total of 53 (11 %) records; of these 20 (4 % of the total) corresponded to recordings mislabeled as one of the predefined modes and 33 (7 % of the total) corresponded to recordings mislabeled as other modes. Thus, the BetterCare® System was able to correctly label the mode 89 % of the time.

## Discussion

Successful clinical decision support systems must provide patient-specific recommendations [14]. To do that, the system requires unequivocal information, at least for some critical variables. For systems advising clinicians about mechanical ventilation, the ventilatory mode being used is critical information that current ventilator communication protocols do not readily provide. The algorithm incorporated in the BetterCare® system correctly identified the ventilatory mode in 433 (89 %) of the 486 hours recorded in 73 patients, with a kappa index of 84.5 % [95 % CI: 80.5–88.4 %]. Landis and Koch [15] proposed the following standards for strength of agreement for the kappa coefficient: ≤0 % = poor, 1–20 % = slight, 21–40 % = fair, 41–60 % = moderate, 61–80 % = substantial and 81–100 % = almost perfect. Although a kappa index of 84.5 % is highly acceptable we still mislabeled 11 % of the cases and continue to improve our recognition algorithms.

Expert systems dealing with mechanical ventilation need to be able to detect ventilatory modes automatically. The number of ventilatory modes has increased dramatically over the last 30 years. Moreover, within each mode many features can be activated that transform the basic mode into what is effectively another mode. To add further confusion, the lack of standards in ventilatory mode naming [11, 12] has resulted not only in different names for the same ventilatory mode but also in different ventilatory modes with the same name. Although manufacturers’ names can be translated into standard names, most communication protocols from mechanical ventilators do not provide information on the ventilatory mode in use. We chose these modes because they accounted for 70 % of the ventilatory time in a recent large international study on the epidemiology of mechanical ventilation [16], and corresponded to 94 % of the modes used in our cohort.

The practical consequences of failing to label a record are different from those of mislabeling a record. If the system cannot determine the ventilatory mode, it cannot provide advice; however, if the system mislabels the mode, it may provide erroneous advice. For example, airway pressure-time profile analysis could provide information about two important determinants of ventilator-induced lung injury: tidal recruitment and overdistention [17]. This analysis derives an index by fitting the central part of the inspiratory airway pressure-time profile to an exponential function. Importantly, however, the index is valid only if the ventilatory mode is VC-CMV and no patient inspiratory efforts are present [17]. Unless both these conditions are met, there is a danger of misinterpretation that could lead to incorrect decisions and place the patient at risk. In the present study, 20 (4 %) records were mislabeled. Of these, eight resulted from problems in differentiating between PC-CMV and VC-CMV_DF_ and four from problems in differentiating between PC-CMV and PC-CSV. Another six records where the actual mode was VC-intermittent mandatory ventilation with pressure support were labeled PC-CSV (two cases) or PC-CMV (four cases).

A critical aspect of our algorithms is to establish if a given parameter is (or is not) stable. If a parameter changed more than 10 %, the algorithm considers it “variable”. To reduce the threshold below 10 % would reduce the number of variable parameters considered constant. Unfortunately, it would also increase the number of constant parameters considered variable: even when these thresholds typically are more precise than required by the ISO standard [18], for delivery of pressures, flows, and volumes by ventilators.

For instance, differentiating PC-CMV from PC-CSV is based on the variability of inspiratory time and distinguishing between VC-CMV_DF_ and PC-CMV, which relies on the variability of airway pressure, the slope of the inspiratory flow curve and tidal volume. However, a fixed ventilatory pattern (or the absence of muscular activity) leads to a scenario in which patients ventilated in PC-CMV will have a constant inspiratory volume and slope of inspiratory flow and patients in VC-CMV_DF_ will have consistent airway pressure. Our analysis of mislabeled records showed that most mistakes occurred in records with very regular ventilatory patterns where the variation in parameters expected to vary was below the tolerance limits we had set. Furthermore, the high inspiratory pressure alarm, for instance, can abort inspiration making parameters expected to be constant become very variable (like inspiratory time in PC-CMV and VC-CMV or tidal volume in VC-CMV).

**Fig. 1:**
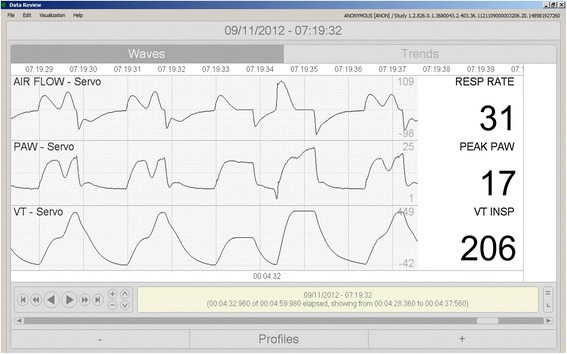
Irregular traces from a patient in PC-CMV. A second inspiratory peak flow in a machine-triggered breath strongly suggests reverse triggering

**Fig. 2:**
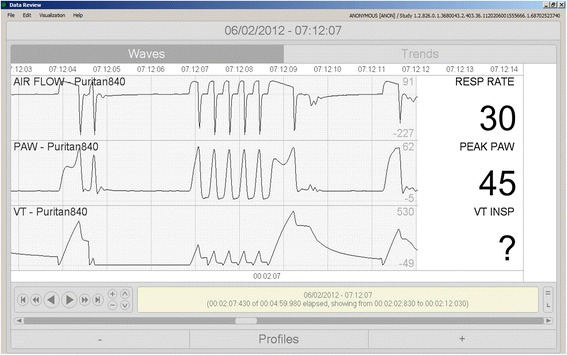
The airway pressure alarm aborts inspiratory cycles, producing highly variable inspiratory times and volumes and making it difficult for the system to correctly identify the mode as VC-CMV

**Fig. 3:**
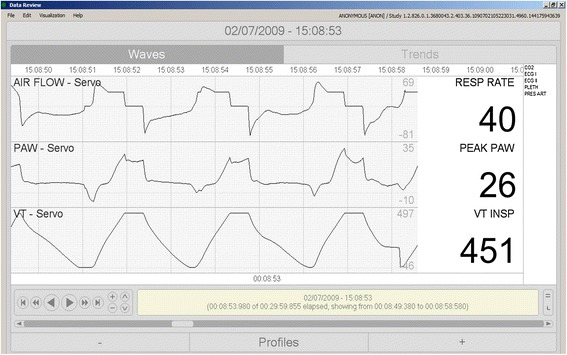
Manufacturer’s non-standard implementation of ventilatory modes. With the goal to increase patient comfort and reduce flow asynchrony, some manufacturers have developed modifications of classic ventilatory modes. For instance, Maquet ventilators in VC-CMV (when airway pressure shows a concavity, inadequate flow to meet patient inspiratory demand), the ventilator opens its demand valve allowing additional gas flow to avoid flow asynchrony leading to an increase in tidal volume potentially violating a lung-protective approach. Beyond its advantages and drawbacks, it causes changes in the inspiratory flow profile and tidal volume that prevent the system from identifying the mode as VC-CMV

A total of 56 (12 %) records were not assigned to one of the modes. In 23 cases the decision was correct, because the actual mode was one of the modes that were outside the scope of the algorithm. In 33 cases, however, the system should have assigned the mode. The main reason for these mistakes was severe patient-ventilator asynchrony that resulted in muddled records that were difficult even for expert physicians to classify (Figs. 1 and 2). Another problem, specific to Servo ventilators, is where in VC-CMV the inspiratory valve opens to mitigate flow asynchrony, causing tidal volume to become highly variable and causing the system to fail to recognize the mode as VC-CMV (Fig. 3).

Determining the ventilator mode is also very important for detecting the occurrence of inspiratory patient-ventilator asynchrony (flow asynchrony, delayed triggering, and patient-ventilator inspiratory time mismatch). In a recent study of patient-ventilator asynchronies in a general population monitoring the entire period of mechanical ventilation, Blanch et al. [10] found that asynchronies are very frequent and are associated with mortality. However, they probably underestimated the prevalence of ineffective inspiratory efforts because the inability to determine the ventilatory mode limited the system’s analyses mostly to the expiratory portion of the ventilatory cycle [9]. Incorporating an algorithm to detect the ventilatory mode in use would enable the entire ventilatory cycle to be analyzed and provide a more complete picture of the problem.

This study’s main strength is that it represents real problems occurring in ICUs. The records analyzed came from a wide variety of real ICU patients with asynchronies, cough, airway secretions, etc. Its main limitation is that it considered a limited set of ventilatory modes. We have not developed algorithms to detect dual modes because their use remains marginal [16]. Moreover, the low number of proportional pressure support records in our database precludes the validation of an algorithm to detect those modes. Thus, the reliability of our algorithms to detect ventilatory modes has been only established for VC-CMV, VC-CMV_DF_, PC-CMV, PC-CSVs PC-CSV_R_+, and CPAP.

## Conclusions

Automatic systems can accurately identify the most commonly used ventilatory modes, thus providing crucial information that enables the entire ventilatory cycle to be analyzed. More data must be collected to validate the algorithm for identifying less frequently used proportional pressure support modes.

## Abbreviations

CPAP, continuous positive airway pressure; DICOM, Digital Imaging and Communication in Medicine; ICU, intensive care unit; NAVA, neurally adjusted ventilatory assist; PACS, picture archiving and communication system; P_AW_, airways pressure; PC-CMV, pressure control-continuous mandatory ventilation or pressure-controlled ventilation; PC-CSV, pressure control-continuous spontaneous ventilation or pressure support ventilation; PC-CSV_R_, spontaneous proportional assist or proportional assist ventilation; PC-CSV_R_+, spontaneous proportional assist or proportional assist ventilation +; TI, inspiratory time; VC-CMV, volume control-continuous mandatory ventilation or volume-controlled ventilation with constant flow; VC-CMV_DF_, volume control-continuous mandatory ventilation with decelerated flow; or volume-controlled ventilation with decelerated flow; VI, variability index; VT, tidal volume

## Additional file

Additional file 1: The database on which this study is based. (XLS 77 kb)

## References

[1] Thibault GE, Mulley AG, Barnett GO, Goldstein RL, Reder VA, Sherman EL, Skinner ER (1980). Medical intensive care: indications, interventions, and outcomes. N Engl J Med.

[2] Pickering BW, Gajic O, Ahmed A, Herasevich V, Keegan MT (2013). Data utilization for medical decision making at the time of patient admission to ICU. Crit Care Med.

[3] Pinsky MR, Dubrawski A (2014). Gleaning knowledge from data in the intensive care unit. Am J Respir Crit Care Med.

[4] Alderman EL, Branzi A, Sanders W, Brown BW, Harrison DC (1972). Evaluation of the pulse-contour method of determining stroke volume in man. Circulation.

[5] Wesseling KH, Jansen JR, Settels JJ, Schreuder JJ (1993). Computation of aortic flow from pressure in humans using a nonlinear, three-element model. J Appl Physiol (1985).

[6] Auler JO, Galas F, Hajjar L, Santos L, Carvalho T, Michard F (2008). Online monitoring of pulse pressure variation to guide fluid therapy after cardiac surgery. Anesth Analg.

[7] Berkenstadt H, Margalit N, Hadani M, Friedman Z, Segal E, Villa Y, Perel A (2001). Stroke volume variation as a predictor of fluid responsiveness in patients undergoing brain surgery. Anesth Analg.

[8] Hofer CK, Muller SM, Furrer L, Klaghofer R, Genoni M, Zollinger A (2005). Stroke volume and pulse pressure variation for prediction of fluid responsiveness in patients undergoing off-pump coronary artery bypass grafting. Chest.

[9] Blanch L, Sales B, Montanya J, Lucangelo U, Garcia-Esquirol O, Villagra A, Chacon E, Estruga A, Borelli M, Burgueno MJ (2012). Validation of the Better Care(R) system to detect ineffective efforts during expiration in mechanically ventilated patients: a pilot study. Intensive Care Med.

[10] Blanch L, Villagra A, Sales B, Montanya J, Lucangelo U, Lujan M, Garcia-Esquirol O, Chacon E, Estruga A, Oliva JC (2015). Asynchronies during mechanical ventilation are associated with mortality. Intensive Care Med.

[11] Chatburn RL, El-Khatib M, Mireles-Cabodevila E (2014). A taxonomy for mechanical ventilation: 10 fundamental maxims. Respir Care.

[12] Chatburn RL, Volsko TA, Hazy J, Harris LN, Sanders S (2012). Determining the basis for a taxonomy of mechanical ventilation. Respir Care.

[13] Halpern NA (2014). Innovative designs for the smart ICU: Part 3: Advanced ICU informatics. Chest.

[14] Payne TH (2000). Computer decision support systems. Chest.

[15] Landis JR, Koch GG (1977). The measurement of observer agreement for categorical data. Biometrics.

[16] Esteban A, Frutos-Vivar F, Muriel A, Ferguson ND, Penuelas O, Abraira V, Raymondos K, Rios F, Nin N, Apezteguia C (2013). Evolution of mortality over time in patients receiving mechanical ventilation. Am J Respir Crit Care Med.

[17] Grasso S, Terragni P, Mascia L, Fanelli V, Quintel M, Herrmann P, Hedenstierna G, Slutsky AS, Ranieri VM (2004). Airway pressure-time curve profile (stress index) detects tidal recruitment/hyperinflation in experimental acute lung injury. Crit Care Med.

[18] ISO 80601-2-12: 2011/Cor 1: 2011. Medical electrical equipment - Part 2-12: Particular requirements for basic safety and essential performance of critical care ventilators. Geneva, Switzerland: International Organization for Standardization (ISO); 2011.

